# Association Between Routine Nephropathy Monitoring and Subsequent Change in Estimated Glomerular Filtration Rate in Patients With Diabetes Mellitus: A Japanese Non-Elderly Cohort Study

**DOI:** 10.2188/jea.JE20180255

**Published:** 2020-08-05

**Authors:** Sachiko Ono, Yosuke Ono, Daisuke Koide, Hideo Yasunaga

**Affiliations:** 1 Department of Biostatistics & Bioinformatics, The University of Tokyo, Tokyo, Japan; 2 Department of General Medicine, National Defense Medical College, Saitama, Japan; 3 Department of Clinical Epidemiology and Health Economics, School of Public Health, The University of Tokyo, Tokyo, Japan

**Keywords:** diabetes mellitus, albuminuria, estimated glomerular filtration rate, diabetic nephropathy, quality indicator

## Abstract

**Backgrounds:**

Current guidelines recommend routine nephropathy monitoring, including microalbuminuria or proteinuria testing, for people with diabetes mellitus; however, its effect in terms of preserving renal function remains unclear. We conducted this study to examine the impact of routine nephropathy monitoring on subsequent changes in estimated glomerular filtration rate.

**Methods:**

We retrospectively identified non-elderly individuals with diabetes mellitus based on the prescription of hypoglycemic agents from a large Japanese database (JMDC, Tokyo, Japan) of screening for lifestyle diseases linked with administrative claims data. We collected data on baseline characteristics including age, sex, comorbidity, and laboratory data. We then examined the association between routine nephropathy monitoring results and change in estimated glomerular filtration rate using a propensity-score inverse probability of treatment weighting method.

**Results:**

Among 1,602 individuals who started taking hypoglycemic agents between 2005 and 2016, 102 (6.0%) underwent routine nephropathy monitoring during the first year of medication for diabetes mellitus. After adjusting for multiple confounding factors, there was no significant difference in subsequent estimated glomerular filtration rate changes between individuals with and without routine nephropathy monitoring (difference in percent change 0.11; 95% confidence interval −2.74 to 2.95).

**Conclusion:**

Routine nephropathy monitoring was not associated with preserved renal function. Current recommendations for the universal application of nephropathy monitoring may have limited value to prevent renal dysfunction in non-elderly individuals with diabetes mellitus.

## INTRODUCTION

Diabetic nephropathy is the most common cause of end-stage renal disease requiring dialysis or kidney transplantation.^1^^,^^2^ The number of people requiring dialysis worldwide is increasing rapidly by approximately 7% annually.^3^ Preserving renal function is, therefore, recognized as key to reducing the economic and clinical burdens of diabetes mellitus.^2^

Cumulative evidence suggests that early interventions, such as intensive glycaemic control^4^^,^^5^ and blood pressure control,^6^^,^^7^ prevent the onset and development of diabetic nephropathy. The importance of this early detection of diabetic nephropathy has led to guidelines recommending routine nephropathy monitoring (microalbuminuria or proteinuria testing, depending on stage of diabetic kidney disease) for all individuals with diabetes.^8^^,^^9^ Consequently, implementation of such testing is used as a quality indicator for diabetes care in many clinical settings and studies.^10^^–^^14^ However, the impact of routine nephropathy monitoring on preventing the progression of diabetic nephropathy remains uncertain, and evidence regarding routine nephropathy monitoring in individuals with diabetes mellitus has been conflicting. Some previous studies indicated that good diabetes care, including routine nephropathy monitoring, did not necessarily improve health outcomes,^15^^,^^16^ while other studies found that adherence to these care processes improved health outcomes, manifested by fewer hospitalizations and diabetes complications.^17^^,^^18^ However, no study to date has directly assessed the association between routine nephropathy monitoring and subsequent renal function in individuals with diabetes mellitus.

We, therefore, investigated the association between routine nephropathy monitoring and subsequent percent changes in renal function among non-elderly individuals with diabetes mellitus, using information from a large Japanese database of annual health surveillance records linked with administrative claims data.

## METHODS

### Data source

We gathered information from the Japan Medical Data Center (JMDC) database for this study. Details of this database are described elsewhere.^19^ The JMDC collects data from more than 60 insurers, with approximately 1.5 million insured individuals in 2013, most of whom are employees of Japanese companies and their family members. More than 95% in the cohort was younger than 65 years of age in 2013. The database includes information on annual health screening and administrative claims data for clinic visits and hospital admissions. Diagnoses are recorded using the International Classification of Diseases, 10th revision (ICD-10) codes and drugs are categorized according to the World Health Organization Anatomical Therapeutic Chemical (WHO-ATC) classification system. The need for informed consent was waived because of the de-identified nature of the data. This study was approved by the institutional review board of The University of Tokyo (approval number 10862-(1)).

### Study population

We identified individuals in the JMDC database aged ≥18 years old who started taking any commercially available hypoglycemic agent (WHO-ATC, A10) at least after 1 year of baseline period, between 2005 and 2016. Individuals without baseline information or follow-up health-screening data and those who had been monitored for albuminuria during the baseline period were excluded. Individuals were also excluded if they had been diagnosed with cancer; any non-diabetic kidney diseases, including glomerular diseases (ICD-10, N00–N08), renal tubulo-interstitial diseases (N10–N16), or other kidney or ureter disorders (N25–29); and those who underwent dialysis during the baseline period.

We extracted and analyzed the following data from the database: age, sex, insured status (the insured or family of the insured), body mass index (BMI), drug prescriptions, diagnoses, medical procedures, concentration of glycated hemoglobin (HbA1c), serum creatinine (SCr), systolic and diastolic blood pressure, hemoglobin (Hb), and institution characteristics (clinic, non-academic hospital, or academic hospital).

### Exposure

We noted if the individuals had been monitored for nephropathy using either microalbuminuria or proteinuria testing at a primary care institution at least once during the first year of medication (1st to 12th months from the first prescription). A primary care institution was defined as an institution where the first hypoglycemic agents were prescribed and where more than two such prescriptions were issued for each patient. Individuals without primary care institutions were excluded from the analyses.

### Outcome measure

**Figure 1.**
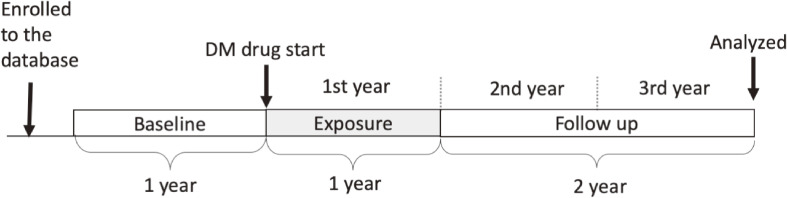
Timeline of the study

We evaluated percent change in estimated glomerular filtration rate (eGFR) over 2 years after nephropathy monitoring (Figure 1), based on previous studies^20^^,^^21^ that reported a strong association between eGFR percent change over 2 years and the development of end stage renal failure later in life. We calculated eGFR based on the patient’s age and SCr measured in the third year (25th to 36th months from the first prescription), using the following formula^22^:

$$\begin{array}{l} \text{eGFR}\;(\text{mL}/\text{min per }1.73\;{\text{m}}^{2})\\ \;=194\times {\text{SCr}}^{-1.094}\times {\text{age}}^{-0.287}\;(\times 0.739\text{ for women}) \end{array}$$

### Statistical analysis

We estimated the average treatment effect in the sample population using the inverse probability of treatment weighting method because nephropathy monitoring is recommended for all people with diabetes mellitus.

We compared baseline characteristics between individuals with and without routine nephropathy monitoring using standardized differences. Standardized differences <0.1 were regarded as a balanced distribution of the covariates. We adjusted for selection bias (ie, individuals with impaired renal function were more likely to be censored by the time of evaluation) by applying inverse probability of censoring weighting using propensity scores to balance characteristics between censored and uncensored individuals. Using this weighting method, censored individuals were weighted for the reciprocal of the propensity score, and uncensored individuals were weighted for the reciprocal of 1 minus the propensity score. Propensity scores for censoring were obtained by fitting a logistic regression model with censoring by the time of evaluation as the dependent variable. Independent variables included nephropathy monitoring, age, sex, BMI, Hb, eGFR, HbA1c, systolic blood pressure, diastolic blood pressure, urinary protein (qualitative), liver disease diagnoses, institution characteristics, and insurance status. Similarly, we adjusted for potential confounding by indication (ie, individuals with impaired renal function were more likely to be tested for albuminuria) by applying inverse probability of treatment (IPT) weighting. Propensity scores for nephropathy monitoring were obtained by fitting a logistic regression model with nephropathy monitoring in the first year as the dependent variable. Independent variables included age, sex, BMI, Hb, eGFR, HbA1c, systolic blood pressure, diastolic blood pressure, urinary protein (qualitative), liver disease diagnoses, institution characteristics, and insurance status. Censored individuals were included only when calculating these two weights, and were not included in the main analyses. For the main analyses, individuals were weighted by total weight (IPT weight × inverse probability of censoring weight). We then fitted a linear regression model with percent change in eGFR over 2 years as the dependent variable and nephropathy monitoring as the independent variable. We used robust standard errors to estimate the variance. We also performed subgroup analysis by repeating the analysis in individuals without hypertension (identified by prescription of antihypertensive or systolic blood pressure ≥140 mm Hg at baseline), given that a previous systematic review suggested that only individuals with diabetes and normal blood pressure might benefit from routine nephropathy monitoring.^23^ We carried out sensitivity analyses by repeating the main analysis restricted to patients with qualitatively negative urinary test results (*N* = 1,287), and setting percent change in eGFR over 1 and 3 years (*N* = 2,419 and *N* = 1,012, respectively) as dependent variables. All statistical analyses were conducted using Stata/MP V.14.2 (StataCorp, College Station, TX, USA) and *P* values <0.05 were considered significant.

## RESULTS

**Figure 2.**
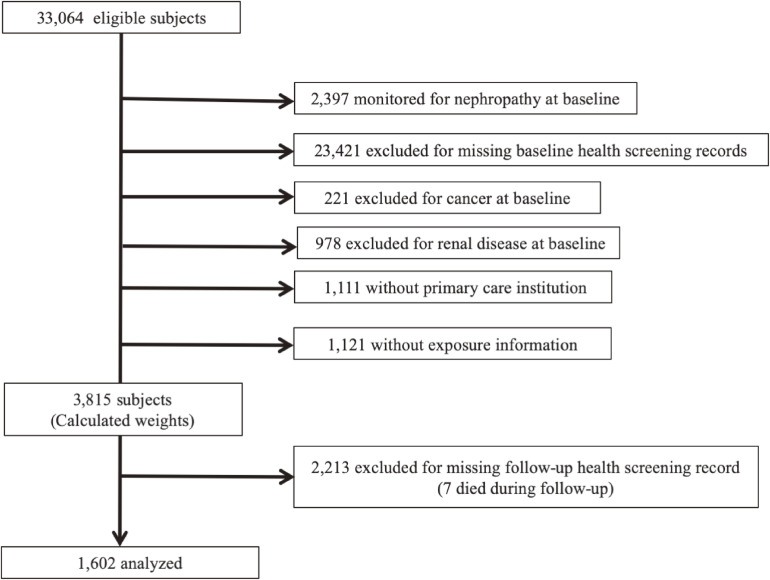
Patient selection

We identified 33,064 people aged ≥18 years who started taking hypoglycemic agents after at least 1 year of baseline period without such prescription (Figure 2). Of these, 2,397 people had been monitored for nephropathy at baseline. We excluded 23,421 because of missing health screening records, 221 because of cancer, and 978 because of renal disease at baseline. We also excluded 1,111 people with no primary care institution and 1,121 with missing exposure information. After calculation of the two weights, we excluded 2,213 people who were censored. Among this censored population, seven individuals died during the follow-up period. Therefore, we analyzed 1,602 individuals.

A total of 102 (6.0%) of the 1,602 individuals were monitored for nephropathy in the first year of taking medication for diabetes mellitus. The demographics and clinical characteristics unweighted for individuals with and without nephropathy monitoring are shown in Table 1. The mean eGFR at baseline was 83.0 (standard deviation [SD], 17.5) mL/min per 1.73 m^2^ for individuals without nephropathy monitoring and 85.9 (SD, 18.1) mL/min per 1.73 m^2^ for those with nephropathy monitoring (standardized difference = 0.162). Individuals with higher HbA1c and higher LDL-cholesterol were more likely to receive nephropathy monitoring. The characteristics of the weighted individuals are shown in eTable 1 and eTable 2. All included variables were balanced between the two groups in the weighted individuals (all standardized differences <0.1). The distribution of total weights is shown in eTable 3.

**Table 1.** Baseline characteristics of individuals with diabetes

	Entire cohort			Normotensive individuals		

Variables	Nephropathy monitoring		Standardized difference	Nephropathy monitoring		Standardized difference
	Without	With		Without	With	
*N*	1,500	102		745	69	
Age, years, mean (SD)	49.6 (7.9)	48.0 (8.0)	0.191	48.4 (7.8)	48.2 (7.4)	0.026
Female	235 (15.7%)	14 (13.7%)	0.055	129 (17.3%)	10 (14.5%)	0.077
The insured (not family member)	1,325 (88.3%)	92 (90.2%)	0.060	656 (88.1%)	62 (89.9%)	0.058
eGFR, mL/min/1.73 m^2^, mean (SD)	83.0 (17.5)	85.9 (18.1)	0.162	84.5 (17.5)	82.9 (17.0)	0.093
HbA1c, %, mean (SD)	7.39 (1.6)	8.1 (1.9)	0.413	7.5 (1.7)	8.2 (2.0)	0.377
BMI, kg/m^2^, mean (SD)	26.9 (4.6)	26.6 (4.2)	0.077	25.9 (4.4)	26.3 (4.4)	0.091
Hb, g/dL, mean (SD)	15.4 (1.3)	15.5 (1.2)	0.084	15.3 (1.4)	15.4 (1.2)	0.077
Systolic blood pressure, mm Hg, mean (SD)	133 (16)	132 (18)	0.054	123 (11)	124 (12)	0.087
Diastolic blood pressure, mm Hg, mean (SD)	82 (11)	82 (12)	0.049	77 (9)	77 (9)	0
LDL-cholesterol, mg/dL, mean (SD)	134 (34)	141 (33)	0.210	137 (35)	144 (32)	0.209
Urinary protein						
−	1,215 (81.0%)	72 (70.6%)	0.245	628 (84.3%)	51 (73.9%)	0.258
±	148 (9.9%)	18 (17.6%)	0.227	64 (8.6%)	11 (15.9%)	0.224
+	97 (6.5%)	9 (8.8%)	0.089	40 (5.4%)	7 (10.1%)	0.176
++	31 (2.1%)	2 (2.0%)	0.008	10 (1.3%)	0 (0.0%)	0.162
+++	9 (0.6%)	1 (1.0%)	0.043	3 (0.4%)	0 (0.0%)	0.090
Liver disease	262 (17.5%)	10 (9.8%)	0.224	98 (13.2%)	8 (11.6%)	0.049
Institution						
Clinic	1,187 (79.1%)	76 (74.5%)	0.109	576 (77.3%)	50 (72.5%)	0.111
Hospital	313 (20.9%)	26 (25.5%)	0.109	169 (22.7%)	19 (27.5%)	0.111
Academic	15 (1.0%)	1 (1.0%)	0.002	2 (0.3%)	0 (0.0%)	0.078

BMI, body mass index; eGFR, estimated glomerular filtration rate; Hb, haemoglobin; HbA1c, glycated hemoglobin; LDL, low-density lipoprotein.

Data are presented as the mean (standard deviation) for continuous variables. Categorical variables are reported as number, *N* (proportion, %).

According to weighting analysis, there was no significant difference in percent change in eGFR over 2 years after the start of hypoglycemic agents between individuals with and without nephropathy monitoring (0.11%; 95% confidence interval, −2.74 to 2.95; *P* = 0.94) (Table 2). The difference was also insignificant among normotensive individuals with diabetes mellitus (−0.54%; 95% confidence interval, −2.68 to 2.14; *P* = 0.26). The findings were similar in the sensitivity analyses restricted to patients with qualitatively negative urinary test results and setting percent change in eGFR over 1 and 3 years as dependent variables (eTable 4, eTable 5, and eTable 6).

**Table 2.** Percent change in eGFR over 2 years based on weighted data for individuals with diabetes

	Nephropathy monitoring		Difference	95% CI	*P*
	With	Without			
Entire cohort	−2.62	−2.73	0.11	−2.74 to 2.95	0.941
Normotensive individuals	−0.54	−2.68	2.14	−1.61 to 5.88	0.263

CI, confidence interval; eGFR, estimated glomerular filtration rate.

Data represent percent change in eGFR (%).

## DISCUSSION

Despite current recommendations and its wide recognition as a quality measure for diabetes care, only 6.0% of non-elderly individuals were monitored for nephropathy during the first year of taking medication for diabetes mellitus. However, after adjusting for multiple confounding factors, we found that nephropathy monitoring had no significant effect on subsequent eGFR changes.

Previous studies demonstrated that early intensive control of diabetes mellitus and hypertension prevented the development of diabetic kidney disease.^4^^–^^7^ Based on these findings, routine nephropathy monitoring has been used as quality measure for diabetes care and is recommended for all individuals with diabetes mellitus, with the aim of early detection of diabetic nephropathy.^8^^,^^9^ One study showed that implementation of tests for HbA1c, lipid, and microalbuminuria was associated with a lower incidence of renal disease among people with diabetes.^18^ Another study also showed an association between process measures including nephropathy monitoring and a lower incidence of all-cause hospitalization.^17^ However, these studies did not assess association between nephropathy monitoring and subsequent renal function directly. A systematic review published in 2005 questioned the value of universal application of nephropathy monitoring because all individuals with diabetes and hypertension benefit from improved glycaemic and blood pressure control.^23^ Identifying urinary albumin may only benefit normotensive individuals with diabetes, because the introduction of antihypertensive agents (currently the only optional medication to reduce albumin excretion) would not otherwise be considered for these individuals.^23^ Our results found no significant association between nephropathy monitoring and subsequent renal function represented by percent change in eGFR over 2 years. This may have been because identifying urinary albumin rarely adds any optional treatment for renal protection in most individuals with diabetes mellitus and hypertension, as pointed out in the above systematic review. Furthermore, the population who might potentially benefit from nephropathy monitoring is quite small; among Asian people with diabetes mellitus, only 0.9% had albuminuria and normal blood pressure.^24^ This small fraction of the population may have made it difficult to detect any positive effect of nephropathy monitoring on subsequent change in outcomes, even in our analysis restricted to normotensive individuals with diabetes mellitus. Another possible explanation for our findings is that intensive control of diabetes mellitus and hypertension is still difficult, even when physicians detect signs of renal function impairment, because intensive glycemic and blood pressure control require the patient’s recognition of the disease and implementation of lifestyle changes.

This study had several limitations. First, the follow-up period may have been too short to observe any decline in renal function. The short follow-up period also prevented ideal analyses (eg, marginal structural model) utilizing exposure as a time-dependent variable. However, to attenuate the former limitation, we analyzed the percent decline in eGFR over 2 years as a surrogate for end-stage renal disease later in life, based on previous research that confirmed a strong association between these two variables.^20^ Second, we may have failed to detect any effect of nephropathy monitoring on change in eGFR because of the small number of individuals with diabetes and normotension who also had albuminuria. However, we focused on the net effect of nephropathy monitoring because this method of nephropathy monitoring is currently recommended for all individuals with diabetes mellitus. Third, the generalizability of this study is limited because most individuals were male workers aged 40–65 years, and we only analyzed those with health screening information. Thus, our results may not be applicable to older populations. Furthermore, because of the universal health care coverage system in Japan, the findings may not be applicable to other countries adopting different health care provision strategies. These factors may have affected the results and conclusions of this study.

Despite these limitations, our findings have important clinical implications, by indicating that the implementation of routine nephropathy monitoring may not improve renal outcomes for all individuals with diabetes mellitus. A previous study reported that a 20% decline in eGFR indicated an increased risk of end stage renal failure later in life, with a hazard ratio of about 2.7 compared with no decline.^20^ The current results showed an approximately 3% decline in eGFR in both individuals with and without nephropathy monitoring. The difference of 0.11% in eGFR might have been too small to attribute any clinical meaning, even if the estimate had been more precise. However, we only assessed eGFR in this study, and we were unable to assess all favorable changes in practice or lifestyle due to a lack of detailed information on individual conditions. The value of nephropathy monitoring as a quality indicator for diabetes care should, thus, be explored further. Moreover, microalbuminuria testing usually costs more than proteinuria testing^25^ (eg, 10 vs 1 United States dollar in Japan). An optimal nephropathy monitoring strategy should be established, especially for individuals who are currently targeted for microalbuminuria testing, rather than recommending the universal application of nephropathy testing.

In conclusion, nephropathy monitoring was not associated with subsequent renal function among non-elderly individuals with diabetes mellitus. Therefore, better adherence to the current recommendations for nephropathy monitoring may not reflect the quality of diabetes care in terms of preserving renal function in non-elderly patients with diabetes mellitus.

## APPENDIX A. SUPPLEMENTARY DATA

eTable 1. Characteristics of inverse probability of treatment weighted patients with diabetes, including those who were censored

eTable 2. Characteristics of inverse probability of censoring weighted patients with diabetes, including those who were censored

eTable 3. Distribution of total weight

eTable 4. Percent change in eGFR over 2 years based on weighted data for individuals with diabetes and qualitative proteinuria test negative

eTable 5. Percent change in eGFR over 1 year based on weighted data for individuals with diabetes

eTable 6. Percent change in eGFR over 3 years based on weighted data for individuals with diabetes
