# Supplementary material for: Association Between Routine Nephropathy Monitoring and Subsequent Change in Estimated Glomerular Filtration Rate in Patients With Diabetes Mellitus: A Japanese Non-Elderly Cohort Study
Source: J Epidemiol. 2020 Aug 5;30(8):326–31. doi: 10.2188/jea.JE20180255 (PMC7348080; doi:10.2188/jea.JE20180255)
Supplement: Supplementary file 1 [file je-30-326-s001.pdf]

**eTable 1.** Characteristics of inverse probability of treatment weighted patients with diabetes, including those who were censored

| | Nephropathy monitoring | | |
|---|---|---|---|
| Variables | Without | With | Standardized difference |
| N | 3,539 | 276 | |
| Age, years, mean (SD) | 50.7 (8.6) | 50.4 (8.4) | 0.043 |
| Female | 677.3 (19.1) | 56.9 (20.8) | 0.041 |
| The insured (not family member) | 3,080.4 (87) | 235.5 (86.1) | 0.028 |
| eGFR, mL/min/1.73 $m^2$, mean (SD) | 82.8 (17.6) | 83.6 (17.1) | 0.046 |
| HbA1c, %, mean (SD) | 7.6 (1.7) | 7.6 (1.6) | 0.043 |
| BMI, kg/$m^2$, mean (SD) | 26.9 (4.8) | 27.1 (5.1) | 0.035 |
| Hb, g/dL, mean (SD) | 15.3 (1.4) | 15.3 (1.3) | 0.032 |
| Systolic blood pressure, mm Hg, mean (SD) | 132 (17) | 133 (19) | 0.055 |
| Diastolic blood pressure, mm Hg, mean (SD) | 82 (12) | 83 (13) | 0.047 |
| LDL-cholesterol, mg/dL, mean (SD) | 135 (35) | 137 (31) | 0.064 |
| Urinary protein | | | |
| – | 2,822.7 (79.7) | 220.7 (80.7) | 0.023 |
| ± | 371.8 (10.5) | 27 (9.9) | 0.021 |
| + | 241.9 (6.8) | 17.9 (6.5) | 0.012 |
| ++ | 84.7 (2.4) | 6.9 (2.5) | 0.008 |
| +++ | 18.6 (0.5) | 1.1 (0.4) | 0.015 |
| Liver disease | 578.1 (16.3) | 44.5 (16.3) | 0.002 |
| Institution | | | |
| Clinic | 2,713 (76.6) | 212.5 (77.7) | 0.025 |
| Hospital | 826.7 (23.4) | 61.1 (22.3) | 0.025 |
| Academic | 38.8 (1.1) | 1 (0.4) | 0.088 |

BMI, body mass index; eGFR, estimated glomerular filtration rate; Hb, haemoglobin; HbA1c, glycated hemoglobin; LDL, low-density lipoprotein.

Data are presented as the mean (standard deviation) for continuous variables. Categorical variables are reported as number, N (proportion, %).

**eTable 2.** Characteristics of inverse probability of censoring weighted patients with diabetes, including those who were censored

| Variables | Uncensored | Censored | Standardized difference |
|---|---|---|---|
| N | 1,602 | 2,213 | |
| Age, years, mean (SD) | 50.6 (8.1) | 50.6 (9.2) | <0.001 |
| Female | 312.8 (19.6) | 428.1 (19.3) | 0.006 |
| The insured (not family member) | 114.6 (7.2) | 159.7 (7.2) | 0.002 |
| eGFR, mL/min/1.73 $m^2$, mean (SD) | 1,383.8 (86.5) | 1,925.7 (86.9) | 0.01 |
| HbA1c, %, mean (SD) | 82.9 (17.5) | 82.9 (17.4) | <0.001 |
| BMI, kg/$m^2$, mean (SD) | 7.6 (1.7) | 7.6 (1.7) | 0.004 |
| Hb, g/dL, mean (SD) | 26.9 (4.6) | 26.9 (4.9) | 0.002 |
| Systolic blood pressure, mm Hg, mean (SD) | 15.3 (1.4) | 15.3 (1.4) | 0.006 |
| Diastolic blood pressure, mm Hg, mean (SD) | 132 (17) | 132 (18) | 0.001 |
| LDL-cholesterol, mg/dL, mean (SD) | 82 (11) | 82 (12) | 0.002 |
| Age, years, mean (SD) | 135 (34) | 135 (35) | 0.003 |
| Urinary protein | | | |
| – | 1,268.8 (79.3) | 1,765.6 (79.6) | 0.008 |
| ± | 168.8 (10.6) | 233.1 (10.5) | 0.002 |
| + | 113 (7.1) | 153.3 (6.9) | 0.005 |
| ++ | 40.5 (2.5) | 53.4 (2.4) | 0.008 |
| +++ | 8.3 (0.5) | 11.6 (0.5) | <0.001 |
| Liver disease | 260 (16.3) | 361 (16.3) | 0.001 |
| Institution | | | |
| Clinic | 1,224.1 (76.5) | 1,697.7 (76.6) | 0.001 |
| Hospital | 375.3 (23.5) | 519.3 (23.4) | 0.001 |
| Academic | 16.5 (1) | 24.1 (1.1) | 0.006 |

BMI, body mass index; eGFR, estimated glomerular filtration rate; Hb, haemoglobin; HbA1c, glycated hemoglobin; LDL, low-density lipoprotein.

Data are presented as the mean (standard deviation) for continuous variables. Categorical variables are reported as number, N (proportion, %).

**eTable 3.** Distribution of total weight

| | N | Mean/Median | SD | Minimum | 1% | 99% | Maximum |
|---|---|---|---|---|---|---|---|
| Entire cohort | 1,602 | 1.0/0.96 | 0.267 | 0.130 | 0.478 | 1.984 | 6.138 |
| Normotensive individuals | 1,127 | 1.0/0.97 | 0.243 | 0.113 | 0.415 | 1.824 | 3.268 |

SD, standard deviation.

Total weight was calculate by inverse probability of censoring weight multiplying by inverse probability of treatment weight in individuals including those who were censored.

**eTable 4.** Percent change in eGFR over 2 years based on weighted data for individuals with diabetes and qualitative proteinuria test negative

| | Nephropathy monitoring | | | | | | |
|---|---|---|---|---|---|---|---|
| | With | Without | Difference | 95% CI | | | P |
| Entire cohort (N=1,287) | -2.46 | -2.63 | 0.17 | -3.07 | to | 3.40 | 0.92 |
| Normotensive individuals (N=679) | -0.44 | -3.06 | 2.62 | -1.75 | to | 6.99 | 0.24 |

CI, confidence interval; eGFR, estimated glomerular filtration rate.

Data represent percent change in eGFR (%).

**eTable 5.** Percent change in eGFR over 1 year based on weighted data for individuals with diabetes

| | Nephropathy monitoring | | | | | | |
|---|---|---|---|---|---|---|---|
| | With | Without | Difference | 95% CI | | | P |
| Entire cohort (N=2,419) | -2.08 | -2.60 | 0.52 | -1.72 | to | 2.76 | 0.65 |
| Normotensive individuals (N=1,228) | -0.04 | -2.49 | 2.45 | -0.29 | to | 5.19 | 0.079 |

CI, confidence interval; eGFR, estimated glomerular filtration rate.

Data represent percent change in eGFR (%).

**eTable 6.** Percent change in eGFR over 3 years based on weighted data for individuals with diabetes

| | Nephropathy monitoring | | | | | | |
|---|---|---|---|---|---|---|---|
| | With | Without | Difference | 95% CI | | | P |
| Entire cohort (N=1,012) | -4.87 | -4.34 | -0.53 | -4.69 | to | 3.64 | 0.80 |
| Normotensive individuals (N=524) | -1.18 | -4.09 | 2.90 | -0.88 | to | 6.69 | 0.13 |

CI, confidence interval; eGFR, estimated glomerular filtration rate.

Data represent percent change in eGFR (%).
